# Complete genome sequence of “*Thiodictyon syntrophicum*” sp. nov. strain Cad16^T^, a photolithoautotrophic purple sulfur bacterium isolated from the alpine meromictic Lake Cadagno

**DOI:** 10.1186/s40793-018-0317-z

**Published:** 2018-05-09

**Authors:** Samuel M. Luedin, Joël F. Pothier, Francesco Danza, Nicola Storelli, Niels-Ulrik Frigaard, Matthias Wittwer, Mauro Tonolla

**Affiliations:** 1 University of Geneva, Sciences III, Department of Botany and Plant Biology, Microbiology Unit, 1211 Geneva, Switzerland; 2 University of Applied Sciences of Southern Switzerland (SUPSI), Department of Environment, Constructions and Design (DACD), Laboratory of Applied Microbiology (LMA), Via Mirasole 22A, 6500 Bellinzona, Switzerland; 3 Federal Office for Civil Protection, Spiez Laboratory, Biology Division, Spiez, Switzerland; 4 Zurich University of Applied Sciences (ZHAW), Institute of Natural Resource Sciences, Environmental Genomics and System Biology Research Group, Wädenswil, Switzerland; 5 University of Copenhagen, Department of Biology, Helsingør, Denmark

**Keywords:** Phototrophic sulfur bacteria, *Chromatiaceae*, Sulfur cycling, Meromictic lake, CRISPR, Okenone

## Abstract

“*Thiodictyon syntrophicum”* sp. nov. strain Cad16^T^ is a photoautotrophic purple sulfur bacterium belonging to the family of *Chromatiaceae* in the class of *Gammaproteobacteria*. The type strain Cad16^T^ was isolated from the chemocline of the alpine meromictic Lake Cadagno in Switzerland. Strain Cad16^T^ represents a key species within this sulfur-driven bacterial ecosystem with respect to carbon fixation. The 7.74-Mbp genome of strain Cad16^T^ has been sequenced and annotated. It encodes 6237 predicted protein sequences and 59 RNA sequences. Phylogenetic comparison based on 16S rRNA revealed that *Thiodictyon elegans* strain DSM 232^T^ the most closely related species. Genes involved in sulfur oxidation, central carbon metabolism and transmembrane transport were found. Noteworthy, clusters of genes encoding the photosynthetic machinery and pigment biosynthesis are found on the 0.48 Mb plasmid pTs485. We provide a detailed insight into the Cad16^T^ genome and analyze it in the context of the microbial ecosystem of Lake Cadagno.

**Electronic supplementary material:**

The online version of this article (10.1186/s40793-018-0317-z) contains supplementary material, which is available to authorized users.

## Introduction

PSB belonging to the family of *Chromatiaceae* are generally found at the interface of aerobic and sulfidic-anaerobic zones that are exposed to sunlight such as stagnant, hypertrophic water bodies, littoral zones and bacterial mats [1]. The genus *Thiodictyon* was first described by Winogradsky in 1888 [2] and comprises two type strains, *Thiodictyon elegans* strain DSM 232^T^ and *Thiodictyon bacillosum* strain DSM 234^T^. “*Thiodictyon syntrophicum**”* sp. nov. strain Cad16^T^ is the proposed type strain of the species “*Thiodictyon syntrophicum**”* [3] within the family of *Chromatiaceae* of the genus *Thiodictyon* [4]. Cultures of strain Cad16^T^ were isolated from the chemocline of the alpine meromictic Lake Cadagno (Ticino, Switzerland). This lake is characterized by high influx of sulfate, magnesium and calcium in the euxinic monimolimnion which favors the formation of a steep chemocline at 10 to 14 m depth [5, 6]. Within this zone a dense population (up to 10^7^ cells per ml in summer) of mainly anaerobic phototrophic sulfur bacteria belonging to the PSB genera *Chromatium**,* *Lamprocystis**,* *Thiodictyon**,* *Thiocystis*, and the GSB *Chlorobium* [7] is responsible for up to 40% of the total CO_2_ fixation measured in Lake Cadagno [8]. Strain Cad16^T^ has been shown to be highly active in CO_2_ fixation both in situ and in vitro [9]*.* Furthermore, aggregation of strain Cad16^T^ with SRBof the genus *Desulfocapsa* has been described [3]. In this publication we describe the first complete genome of strain Cad16^T^ providing details especially on CO_2_ fixation, sulfur metabolism and on CRISPRs. The sequencing of strain Cad16^T^ is part of a larger sequencing project that includes the key species of the microbial community from the anoxic layers of Lake Cadagno.

## Organism information

### Classification and features

**Fig. 1:**
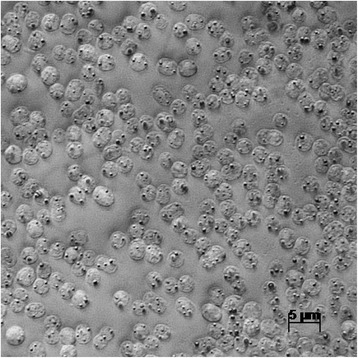
Phase-contrast photomicrograph of “T. syntrophicum” sp. nov. strain Cad16^T^. The elementary sulfur globule inclusions are visible as black dots within the cell

**Fig. 2:**
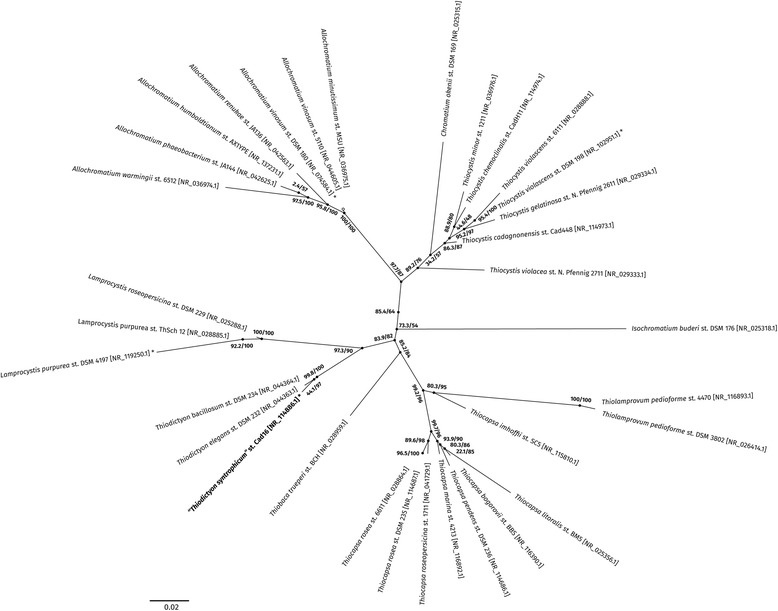
Phylogenetic tree indicating the position of “T. syntrophicum” sp. nov. strain Cad16^T^ relative to other genomes sequenced within the family *Chromatiaceae*. The tree was inferred from 16S rRNA sequences (≥ 1300 bp) using the maximum likelihood algorithm of the *IQ-TREE* software [62]. 1000 bootstrap replicates were performed. Numbers at the nodes indicate the SH-aLRT support (%) and ultrafast bootstrap support (%), respectively. Asterisk mark strains with complete genome sequences available. Open circles as node shapes indicate bootstrap support values below 50%

Strain Cad16^T^ is Gram-negative, the cells are oval-sphere shaped and 1.4–2.4 μm in diameter, non-motile, vacuolated and contain BChl *a*. Isolate Cad16^T^ can grow as single cells, as well as in cell aggregates with up to 100 cells contained in EPS layer (Fig. 1). It was isolated from the chemocline of Lake Cadagno in a depth of 10–14 m where it grows in a non-obligate mutualistic association with sulfur-reducing bacteria of the genus *Desulfocapsa* [10]*.* Based upon morphology and partial 16S rRNA sequence analysis, the strain Cad16^T^ was classified as a member of the genus *Thiodictyon* within the family *Chromatiaceae* before [10]*.* Figure 2 shows the phylogenetic placement of strain Cad16^T^ (complete 16S rRNA sequence) in a 16S rRNA based maximum likelihood phylogenetic tree. The closest relatives of isolate Cad16^T^ are *T. bacillosum* DSM 234^T^ and *T. elegans* DSM 232^T^ with 99% sequence identity (partial 16S rRNA sequences). A comparison of the strain Cad16^T^ core genome with other whole genome sequenced PSB confirmed the phylogenetic placemant (Additional file 1: Figure S1).

Strain Cad16^T^ was anaerobically grown in Pfennigs medium [11], containing per liter: 0.25 g KH_2_PO_4_, 0.34 g NH_4_Cl, 0.5 g MgSO_4_·7H_2_O, 0.25 g CaCl_2_·2H_2_O, 0.34 g KCl, 1.5 g NaHCO_3_, 0.5 ml trace element solution SL_10_, and 0.02 mg vitamin B_12_ with 2 mM acetate in 100 mL serum bottles with rubber stoppers. The medium was prepared in a 2 l bottle with a N_2_/CO_2_ (80%/20%) gas phase. The medium was then reduced with 0.3 g l^− 1^ Na_2_S·9H_2_O (1.10 mM final concentration) and adjusted to a pH of 7.2. Cultures were incubated at 20–23 °C under photoheterotrophic conditions with 6 h light/dark photoperiods with a 40-W tungsten bulb placed at a distance of 60 cm from the cultures (ca. 10 μE m^− 2^ s^− 1^).

Different electron donors and carbon substrates were tested under phototautotrophic conditions by Peduzzi et al. [3, 10]. Photolithoautotrophic growth was observed under anoxic conditions with hydrogen sulfide, thiosulfate and elemental sulfur as electron donors. Thereby, elemental sulfur is stored within the periplasma as intermediate oxidation product (Fig. 1). The carbon sources acetate, butyrate, ethanol, formate, fructose, fumarate, glucose, glycerol, lactate, malate, propanol, propionate, pyruvate and succinate were added at 5 mM concentration, respectively. Strain Cad16^T^ was observed to assimilate only acetate, pyruvate and fructose in the presence of sulfide and bicarbonate. Strain Cad16^T^ was additionally tested for chemolithoautrophic growth with bicarbonate under a headspace atmosphere containing 5% O_2_, 10% CO_2_ and 85% N_2_, in the dark. Growth was observed with 0.02% hydrogen sulfid and 0.07% thiosulfate, or with 0.07% sulfide only, respectively. The pigments responsible for the purple-red color of strain Cad16^T^ were analysed spectrometrically in vivo by Peduzzi et al. [3]. Local absorption maxima at 833 nm, 582 nm and 374 nm gave evidence for the presence of BChl *a*, and at 528 nm for the carotenoid okenone, respectively [10].

A further characterization of strain Cad16^T^ can be found in Table 1.

**Table 1:** Classification and general features of “*T. syntrophicum”* sp. nov. strain Cad16^T^ according to the MIGS recommendations [65]

MIGS ID	Property	Term	Evidence code^a^
	Classification	Domain *Bacteria*	TAS [3, 59]
		Phylum *Proteobacteria*	TAS [3]
		Class *Gammaproteobacteria*	TAS [3]
		Order *Chromatiales*	TAS [3]
		Family *Chromatiaceae*	TAS [3]
		Genus *Thiodictyon*	TAS [2, 60]
		Species “”	TAS [3]
		Strain: Cad16^T^	TAS [3]
	Gram stain	Negative	TAS [3]
	Cell shape	Coccus	TAS [3]
	Motility	Non-motile	TAS [3]
	Sporulation	No	NAS
	Temperature range	5–25 °C	TAS [3]
	Optimum temperature	20–23	TAS [3]
	pH range; Optimum	6.8–7.5	TAS [3]
	Carbon source	CO_2_, acetate, pyruvate, fructose	TAS [3]
MIGS-6	Habitat	Fresh water, alpine meromictic lake	TAS [3]
MIGS-6.3	Salinity	Not determined	NAS
MIGS-22	Oxygen requirement	Aerotolerant	TAS [3]
MIGS-15	Biotic relationship	Free-living	TAS [3]
MIGS-14	Pathogenicity	Non-pathogen	NAS
MIGS-4	Geographic location	Switzerland, Ticino	TAS [3]
MIGS-5	Sample collection	08.28.2001	TAS [3]
MIGS-4.1	Latitude	46°33’ N	TAS [3]
MIGS-4.2	Longitude	8°43′ E	TAS [3]
MIGS-4.4	Altitude	1923 m	TAS [3]

^a^Evidence codes – *IDA* Inferred from Direct Assay, *TAS* Traceable Author Statement (i.e., a direct report exists in the literature), *NAS* Non-traceable Author Statement (i.e., not directly observed for the living, isolated sample, but based on a generally accepted property for the species, or anecdotal evidence). These evidence codes are from the Gene Ontology project [10]

**Fig. 3:**
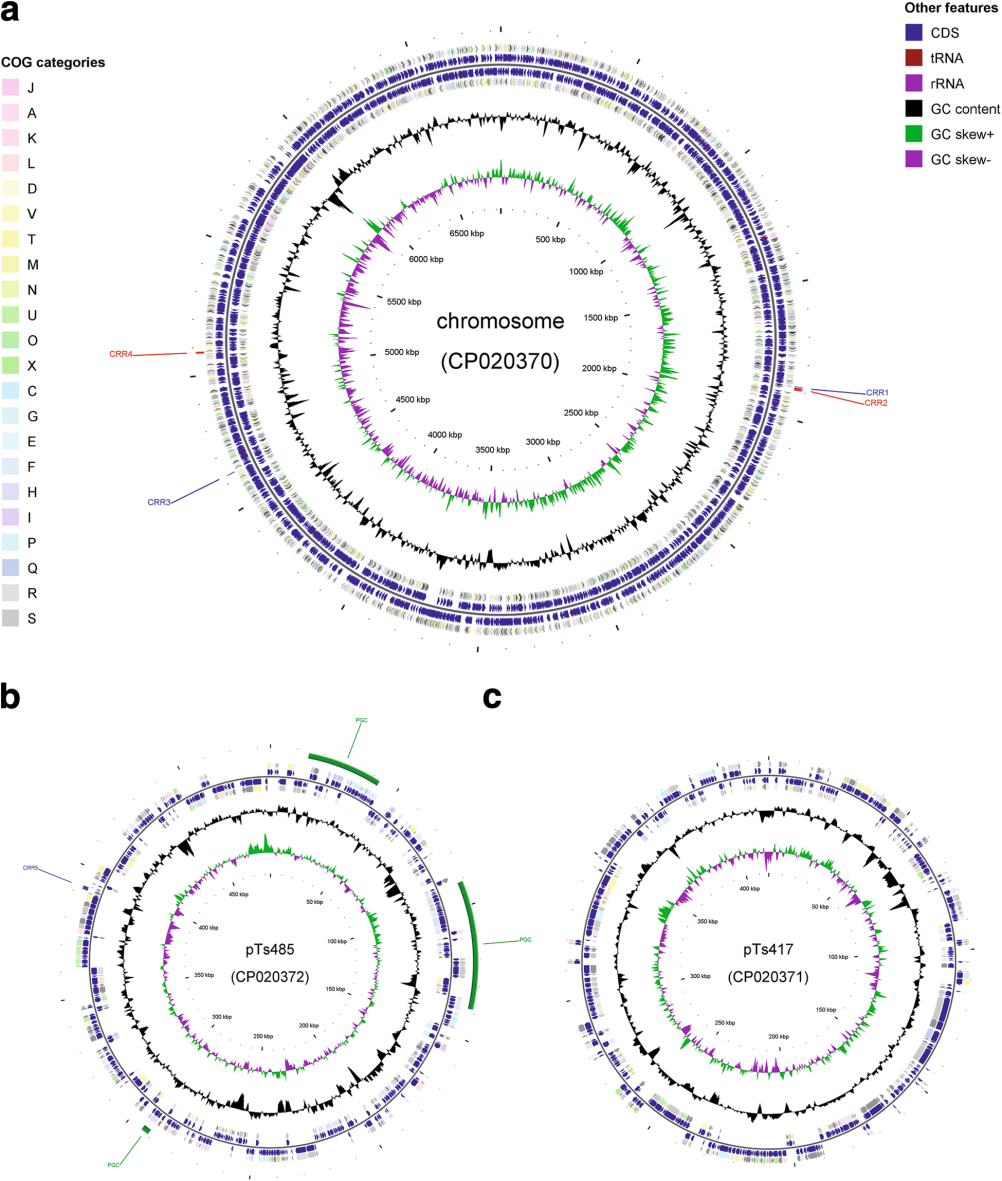
Schematic representation of the complete genome sequence of “Thiodictyon syntrophicum” sp. nov. strain Cad16^T^ and gene classification according to the COG criteria. The genome comprises one circular chromosome (**a**) and two circular plasmids, pTs485 (**b**) and pTs417 (**c**). Beginning with the outermost circle, tracks are shown in the order of: (1) predicted PGC on pTs485 (green), (2) CRISPR arrays on the chromosome and pTs485 (alternating in blue and red) (3) protein coding genes on forward strand colored according to COG categories, (4) CDS (blue), tRNA (orange) and rRNA (violet) on forward strand, (5) CDS (blue), tRNA (orange) and rRNA (violet) on reverse strand, (6) protein coding genes on reverse strand colored according to COG categories, (7) GC content (black), (8) positive and negative GC skew (green and purple, respectively) and (9) genome region by kbp. GCView [63] was used to create this genome map

A circular representation of the genome sequence and annotation according to the COG criteria is shown in Fig. 3.

## Genome sequencing information

### Genome project history

Sampling was done in August 2001 using a Friedinger-type bottle on Lake Cadagno. Subsequent isolation and cultivation of strain Cad16^T^ was done in Pfennig’s medium I [11]. gDNA was isolated in November 2014 and sequencing was performed in January 2015. Raw data was assembled in with the SMRTview assembly platform and annotated using the NCBI Prokaryotic Genome Annotation Pipeline. Completeness of the isolate Cad16^T^ sequence was verified using the 31 single copy genes of the Amphora Net analysis platform [12].

The genome sequence was deposited in GenBank under BioProject PRJNA354524, with the accession numbers CP020370-CP020372. The key elements of the genome studied are listed in Table 2.

**Table 2:** Project information for “T. syntrophicum*”* sp. nov. strain Cad16^T^

MIGS ID	Property	Term
MIGS 31	Finishing quality	Complete
MIGS-28	Libraries used	SMRT 10 kb (BluePippin size selection)
MIGS 29	Sequencing platforms	PacBio RS II
MIGS 31.2	Fold coverage	200×
MIGS 30	Assemblers	HGAP2
MIGS 32	Gene calling method	GeneMarkS+, software revision: 4.1
	Locus Tag	THSYN
	GeneBank ID	CP020370.1, CP020371.1, CP020372.1
	GeneBank Date of Release	07/12/2017
	GOLD ID	Gp0131589
	BIOPROJECT	PRJNA354524
MIGS 13	Source Material Identifier	NA
	Project relevance	Environmental

### Growth conditions and genomic DNA preparation

Strain Cad16^T^ was anaerobically grown in Pfennigs medium [11] Cells were collected by centrifugation for 15 min at 10,600 g. DNA was extracted using phenol/chloroform/isoamylalcohol solution (25:24:1, *v*/*v*, Sigma, Buchs, Switzerland) following the protocol provided by Pacific Biosciences [13] in combination with phase lock gels (VWR International). gDNA was purified using AMPure beads (Agencourt, Beckman Coulter Life Sciences, Indianapolis, USA) following the E2612 protocol form New England Biolabs [14]. Purity of the DNA was tested using the Qbit U*V*/VIS absorption reader (Thermo Fisher Scientific, Rheinach, Switzerland).

### Genome sequencing and assembly

The library construction and genome sequencing was done on the Pacific Biosciences RS II platform at the Functional Genomic Center Zurich, Zurich, Switzerland. A 10 kb SMRTbell library was constructed using the DNA Template Prep Kit 1.0 (Pacific Biosciences, Menlo Park, USA). SMRTbell template fragments over 10 kb length were used for creating a SMRT bell-Polymerase Complex with P6-C4 chemistry (Pacific Biosciences) according to the manufacturer instructions.

Four SMRT cells v3.0 (Pacific Biosciences) for PacBio RS II chemistry were used for sequencing. Separate sequencing quality reports for all four cells were created through the SMRT portal software.

The SMRT web portal was used for genome assembly with the RS_HGAP_Assembly.2 pipeline from the SMRT Analysis 2.3 server. The polished assembly consists of 153 scaffolds with a mean coverage of 175× and a N50 value of 6,849,178. Thereof, three scaffolds were distinctly longer (6.85, 0.50 and 0.43 Mb, respectively) and showed a coverage greater than 200×, whereas mean coverage dropped below a value of 50× for the remaining 150 scaffolds.

These three scaffolds showed self-similar ends in dot-plot graphs and could be circularized manually.

The genome was manually corrected for SNPs using MiSeq Illumina 300-bp paired-end reads from previous sequencing (unpublished data, N. Storelli, J.F. Pothier, M. Tonolla).

### Genome annotation

NCBI Prokaryotic Genome Annotation Pipeline (Annotation Software revision 4.1) NCBI Prokaryotic Genome Annotation Pipeline (Annotation Software revision 4.1) was used for gene calling and gene annotation. To identify CRISPR-Cas sequences the CRISPRfinder server was used [15]*.* The Pfam-A v29 database was used to predict Pfam domains [16]. Transmembrane domains were predicted with the webserver based TMHMM2 program [17] and signal peptides were predicted with SignalP 4.1 server [18].

## Genome properties

The complete genome of strain Cad16^T^ comprises one circular chromosome (6,837,296 bp) and two circular plasmids pTs485 (484,824 bp) and pTs417 (416,864 bp) (Table 3). The average GC content for the chromosome, and plasmids pTs485 and pTs417, is 66.28%, 65.59 and 65.97%, respectively. A total of 6601 coding sequences were predicted. Thereof, 6237 were predicted to encode proteins whereas six rRNA, 49 tRNA and four ncRNA sequences were predicted. A putative function is assigned for 3471 (46.57%) protein encoding genes (Table 4). The classification of genes into COGs functional categories is given in Table 5. The replicons pTs485 and pTs417 could be made circular, have their own origin of replication each, but do not contain any RNA or house-keeping genes. Therefore, to our understanding, both pTs485 and pTs417 fulfill the plasmid definition.

**Table 3:** Summary of genome of “T. syntrophicum*”* sp. nov. strain Cad16^T^: one circular chromosome and two circular plasmids

Label	Size (Mb)	Topology	INSDC identifier	RefSeq ID
Chromosome	6.84	Circular	CP020370	NA
pTs417	0.42	Circular	CP020371	NA
pTs485	0.49	Circular	CP020372	NA

**Table 4:** Genome statistics for the “T. syntrophicum*”* sp. nov. strain Cad16^T^ genome

Attribute	Value	% of Total
Genome size (bp)	7,738,984	100.00
DNA coding (bp)	6,663,511	86.10
DNA G + C (bp)	5,124,386	66.22
DNA scaffolds	3	100.00
Total genes	6601	100.00
Protein coding genes	6237	94.49
RNA genes	59	0.89
rRNA genes	6	0.09
tRNA genes	49	0.74
ncRNA genes	4	0.06
Pseudo genes	305	4.62
Genes in internal clusters	NA	NA
Genes with function prediction	2737	41.46
Genes assigned to COGs	3157	47.83
Genes with Pfam domains	4675	70.82
Genes with signal peptides	436	6.61
Genes with transmembrane helices	1185	17.95
CRISPR repeats	5	–

*NA* not applicable

**Table 5:** Number of genes associated with general COG functional categories of the genome of “T. syntrophicum*”* sp. nov. strain Cad16^T^. The percentage is set relative to the total number of protein coding genes in the genome

Code	Value	% age	Description
J	210	3.37	Translation, ribosomal structure and biogenesis
A	1	0.02	RNA processing and modification
K	144	2.31	Transcription
L	276	4.43	Replication, recombination and repair
B	0	0.00	Chromatin structure and dynamics
D	42	0.67	Cell cycle control, Cell division, chromosome partitioning
Y	0	0.00	Nuclear structure
V	186	2.98	Defense mechanisms
T	297	4.76	Signal transduction mechanisms
M	274	4.39	Cell wall/membrane biogenesis
N	10	0.16	Cell motility
Z	0	0.00	Cytoskeleton
W	0	0.00	Extracellular Structures
U	62	0.99	Intracellular trafficking and secretion
O	226	3.62	Posttranslational modification, protein turnover, chaperones
X	97	1.56	Energy production and conversion
C	245	3.93	Energy production and conversion
G	122	1.96	Carbohydrate transport and metabolism
E	167	2.68	Amino acid transport and metabolism
F	49	0.79	Nucleotide transport and metabolism
H	135	2.16	Coenzyme transport and metabolism
I	93	1.49	Lipid transport and metabolism
P	184	2.95	Inorganic ion transport and metabolism
Q	29	0.46	Secondary metabolites biosynthesis, transport and catabolism
R	308	4.94	General function prediction only
S	1522	24.40	Function unknown
No COG	1543	24.74	Not in COGs
Multi COG	320	5.13	Multiple COG assignments

### Extended insights from the genome sequence

#### Phototrophy

PSB typically transform light energy into chemical energy with the membrane bound type 2 photochemical reaction center. The chromosome of strain Cad16^T^ encodes the core antenna proteins LH1, subunits PufA and PufB (THSYN_31145 and THSYN_31140), and the regulatory protein PufQ (THSYN_31110) upstream to the reaction center genes composed of reaction RC subunits PufL, PufM, and PufC (THSYN_31125–31,135). Additional two copies of subunits LH2 alpha and beta (THSYN_31115 and THSYN_31120), respectively, are encoded further downstream, as well as pairwise in two other clusters (THSYN_30995/31005/31030/31040 and THSYN_31000/3100531010/31035/31045), similar as described for the PSB *Allochromatium vinosum* DSM 180^T^ [19]. The photosynthetic reaction center H subunit PuhA (THSYN_31405) and PucC (THSYN_31410) are clustered upstream with genes encoding RC-LH1 auxiliary proteins (THSYN_31390–31,400). Furthermore, a homologousHiPIP (THSYN_25970) is found in strain Cad16^T^. It may function as the main electron donor to the photosynthetic reaction center similar as in *A. vinosum* [20].

The absorption spectrum of strain Cad16^T^ shows strong absorption peaks at 374 nm, 582 nm and 833 nm which are characteristic for BChl *a* [10]. The genes for the complete enzymatic pathway from protoporphyrin to chlorophyllide, and further to BChl *a* (THSYN_31090–31,105, THSYN_31375, THSYN_31385, THSYN_31415–31,445, THSYN_31555, THSYN_32265–32,270), are clustered on pTs485. BChl *a* formation is thereby catalyzed by an anaerobic type of the Mg-protoporphyrin IX monomethyl ester oxidative cyclase (ChlE) (THSYN_31385) and a light independent protochlorophyllide reductase complex (ChlLNB) (THSYN_31420–31,430) in strain Cad16^T^.

Strain Cad16^T^ produces okenone as its sole carotenoid [10] and Crt proteins involved in carotenoid biosynthesis are found on pTs485. The complete synthesis of this keto-carotenoid is mediated through two novel types of carotenoid ketolases, the C-4/4′ ketolase CruO (THSYN_31065) and the oxygen dependent CruS bifunctional desaturase (THSYN_31070) [21]. The characteristic χ-ring of okenone is introduced through the key enzymes CrtY and CrtU (THSYN_31055 and THSYN_31050) [21, 22].

Remarkably, most of the proteins involved in photosynthesis are encoded on plasmid pTs485, forming a PGC (Fig. 3) [23]. The highly modular character of the *pufLM* and *pufC* genes of α, β and γ-proteobacteria has been demonstrated previously [24, 25]. To our knowledge, this is the first description of a PGC being localized on a plasmid in a PSB species. Interestingly, the gene cluster is similarly organized as in the γ-proteobacterium *Congregibacter litoralis* strain KT71^T^ and as in members from the α-proteobacteria families *Rhodobacteraceae* and *Rhodospirillaceae*, respectively.

#### Sulfur metabolism

For the photoautotrophic process of CO_2_ assimilation in PSB, electrons derived from the oxidation of reduced sulfur compounds, are transferred to electron carriers NAD(P)^+^ and ferredoxin through light energy. During photolithoautotrophic growth under anaerobic conditions, strain Cad16^T^ uses electrons from the oxidation of sulfide, thiosulfate and elemental sulfur as reducing equivalents [3]. Strain Cad16^T^ can use thiosulfate as an electron source during phototrophic growth [3]. No homologous genes for the thiosulfate oxidizing multi-enzyme complex SoxAX, could be found in the strain Cad16^T^ genome. However, *soxB* (THSYN_26690) and clustered genes encoding SoxYZ (THSYN_09005–09010) that binds thiosulfate were identified in the genome. Remarkably, this gene combination is found in several genome sequenced *Ectothiorhodospiraceae*. In contrast to the PSB *A. vinosum* DSM 180^T^ [26], no homologous sequence for the tetrathionate-forming thiosulfate dehydrogenase TsdA was found. However, a c4 cytochrome type TsdB homolog (THSYN_17090) was identified. Due to this unusual combination of genes involved in thiosulfate oxidation, further studies are needed to elucidate the thiosulfate oxidation pathways in strain Cad16^T^.

Initial sulfide and thiosulfate oxidation is immediately followed SGB formation in strain Cad16^T^ (Fig. 1). In strain Cad16^T^ the SGB structure is mediated through envelope SGP homologues to SgpA and SgpB (THSYN_20250 and THSYN_05960) from “*Thioflavicoccus mobilis**”* and *Thiocystis violascens*, respectively. The sequence of SgpC (THSYN_11025) shows homology to *Marichromatium* species SgpC/CV3*.* Predicted signal peptides suggest export of for all three SGP proteins into the periplasm in Cad16^T^, as proposed for *A. vinosum* DSM 180^T^ [27].

Moreover, the genome of strain Cad16^T^ encodes the membrane-bound sulfide: quinone oxidoreductases SqrD (THSYN_04215) and SqrF (THSYN_09305). These are possibly involved in the oxidation of sulfide in the periplasm.

The mode of sulfur transport across the inner membrane is not known for PSBs [28]. Organic persulfides such as glutathione or glutathione amide persulfide are proposed as possible candidates. In a next step, the rhodanese-like protein Rhd transfers the sulfur from the persulfide-carrier to the TusA protein in the cytoplasm. The further oxidation steps from sulfur to sulfite are typically mediated through the reverse acting *dsr* genes in PSB [29]. The strain Cad16^T^ genes in the *dsr* cluster (THSYN_22480, THSYN_22490–22,545) are arranged in a highly conserved organization similar to *A. vinosum* DSM 180^T^, only missing *dsrS* that is non-essential for sulfur oxidation [30]. The DsrEFH complex mediates persulfate transfer from TusA onto DsrC. The persulfurated form of DsrC is then substrate for the cytoplasmic reverse-acting dissimilatory sulfite reductase DsrAB that catalyzes the formation of sulfite. Finally, DsrMKJOP complex reduces DsrC [30].

The genome harbors three additional sulfur relay proteins similar to DsrC (THSYN_09485, THSYN_18820 and THSYN_22565) that could function as TusA homologues. In *A. vinosum* DSM 180^T^ DsrC is able to bind DNA upstream the *dsr* cluster [31].

In strain Cad16^T^, *soeABC* (THSYN_16370–16,380) encode the sulfur-iron molybdoprotein complex that further oxidizes sulfite to sulfate on the cytoplasmic site of the membrane [32]. Alternatively, strain Cad16^T^ oxidizes sulfite via APS by APS-reductase AprBA (THSYN_16395 and THSYN_16400) and ATP sulfurylase Sat (THSYN_16390), as in other PSB [33, 34]. Thereby, the membrane-bound QmoABHdrCB-complex [35] (THSYN_16425–6440) possibly functions as an electron acceptor for the AprAB reductase complex since no *aprM* homolog was found in the strain Cad16^T^ sequence. For the extra-cytoplasmic export of the final oxidation product sulfate, a SulP sulfate permease (THSYN_14085) homolog to *A. vinosum* DSM 180^T^ is encoded in the strain Cad16^T^ sequence.

Hydrogen uptake and consumption has been shown to be linked to sulfur metabolism in *Thiocapsa roseopersicina* BBS [36, 37]. Thereby, electrons from hydrogen oxidation in the periplasm by the hyn-type hydrogenase HydSL could be transferred via the Isp membrane complex to the disulfide bound to DsrC. In *A. vinosum* DSM 180^T^*,* transcription of *isp1* and *isp2* encoding the Isp hydrogenase subunits is upregulated during growth on sulfide [38]. The Isp complex is composed of two subunits, Isp1 and Isp2, that contain similar catalytic domains as DsrM and DsrK, respectively. Similarly, homologous Isp1 and Isp2 proteins (THSYN_28105 and THSYN_28100) may link sulfur to hydrogen metabolisms in strain Cad16^T^. In accordance, an increase in the sulfide concentration was observed while SGB were consumed by strain Cad16^T^ during incubation in the dark (unpublished results, F. Danza).

Additionally, other [NiFe]-hydrogenases of the Hox and Hup type (THSYN_22655, THSYN_22660 and THSYN_28115) are found in the sequence that could mediate light-dependent H_2_ evolution as proposed for *T. roseopersicina* [39, 40].

The Cad16^T^ genome also harbors *cys* genes (THSYN_05020–05035) that are probably involved in sulfate assimilation under sulfur-limiting conditions. Furthermore, the genome also encompasses genes encoding the CydDC (THSYN_18930 and THSYN_18935) ATP-driven cysteine transport proteins [41].

#### Autotrophic growth

In PSB, CO_2_ fixation is essentially achieved through the reductive pentose phosphate also known as the CBB cycle. In accordance, the strain Cad16^T^ genome harbors the complete CBB enzymatic pathway. On the chromosome, the dimeric RuBis-CO form II (THSYN_13250) clusters with RuBis-CO activation protein subunits CbbR, CbbQ and CbbO, (THSYN_13245, THSYN_13255 and THSYN_13285). Interestingly, small and large RuBis-CO subunits form I (THSYN_29475 and THSYN_29480) cluster together with carboxysome shell and auxiliary proteins on plasmid pTs417 (THSYN_29485–29,520 and THSYN_29530–29,535). The carboxysome may allow efficient photoassimilation across varying CO_2_ concentrations as proposed for *A. vinosum* DSM 180^T^ [42]. Previous studies showed different expression regulation for RuBis-CO type I and type II genes from Cad16^T^ suggesting that only the type II is involved in the process of CO_2_ fixation [8]. Interestingly, the plasmid pTs485 also harbors a RuBis-CO -like protein form III gene (THSYN_31160) upstream the PGC.

The missing sedoheptulose-1,7-bisphosphatase SBP is possibly bypassed by via the fructose-1,6-bisphosphatase (THSYN_25630). The genes *gltA* citrate synthase (THSYN_12620), *fumA* fumarate hydratase (THSYN_24360) and *sucCD* succinyl-CoA ligase (THSYN_00880 and THSYN_00885) that are essential for the TCA cycle, and isocitrate lyase (THSYN_16275) and malate synthase (THSYN_15655) that are essential for the glyoxylate cycle, respectively, are identified in the strain Cad16^T^ sequence. Recently a proteomic study about the capacity of Cad16^T^ to fix CO_2_ in the dark suggested the presence of a particular archael DC/HB cycle [42]. However, nofurther genes coding for this DC/HB cycle were found. Also a complete set of genes coding for polyhydroxyalkanoic acid synthase PhaC (THSYN_06910) and poly-(3-hydroxybutyrate) depolymerase PhaE (THSYN_06905) are found in the strain Cad16^T^ genome.

Strain Cad16^T^ additionally encodes genes necessary for glycogen polymerisation. The glucose 1-phosphate adenylyltransferase GlgC (THSYN_00810), the glycogen synthase GlgA (THSYN_11615) and the 1,4-alpha-glucan branching enzyme GlgB (THSYN_00805) allow the synthesis of glycogen.

Interestingely, strain Cad16^T^ also has the potential to produce the storage compound cyanophycin normally found in *caynobacteria* [43], since the two subunits of the enzyme cyanophycin synthetase (THSYN_26990 and THSYN_26995) are found.

Togheter, these finding provide genetic evidence for the high carbon fixation potential of strain Cad16^T^ in the dark [8, 44].

Anaerobic Fe(II)-oxidation was described for other *Thiodictyon* strains [45, 46] and evidence of cryptic in situ iron cycling has been demonstrated recently [47]. In accordance with these findings, we found *cbb3* type terminal cytochrome C oxidases (THSYN_06760–08775) possibly involved in Fe(II) driven carbon fixation in strain Cad16^T^ genome.

Strain Cad16^T^ grows chemoautotrophically under microaerobic conditions (5% O_2_) with sulfide, thiosulfate, or sulfide only [3], as also observed in other PSB in vitro studies with *Lamprocystis purpurea* [10, 48], *Thiocystis violacea* and *A. vinosum* [49]*.* In situ, strain Cad16^T^ is possibly exposed to low concentration of oxygen produced by oxygenic microbiota at the mixolimnion-chemocline interface [8]. Accordingly, we observe genes encoding *sod*-type superoxide dismutases (THSYN_20405 and THSYN_22720), as well as *fnr* and *fur-*type transcriptional regulators involved in peroxide stress response. In situ, strain Cad16^T^ is possibly exposed to oxygen produced by oxygenic microbiota at the mixolimnion-chemocline interface [8].

#### Nitrogen metabolism

Furthermore, with the genes encoding NifB (THSYN_03975), NifD (THSYN_08880), NifH (THSYN_08885), NifK (THSYN_08875), NifT (THSYN_08870) NifW, NifZ and NifM (THSYN_10720, THSYN_10725 and THSYN_10730), NifX (THSYN_21435) and NifL (THSYN_24590) strain Cad16^T^ could possibly fix nitrogen. Genes encoding the multisubunit urease UreDEFG (THSYN_03745, 03750, 03760 and 03765) and the urea transporter UrtABCDE (THSYN_07940–07955, 03760, 07975) indicate the possible utilisation of urea.

#### Transmembrane transport proteins

Several membrane transport genes were found in the strain Cad16^T^ genome, including protein secretion system Type II, genes encoding the TAT pathway and several TRAP transporter genes, as well as genes encoding Ton-Tol type and ABC-type transporter complexes. Additionally, a complete TSS4 pilus machinery is encoded in six clusters dispersed on the strain Cad16^T^ chromosome. Notably, also structural components of TSS6 secretion system are found in two clusters on the chromosome (THSYN_11395–11,410) and on pTs485 (THSYN_32540-THSYN_32580). Two effector proteins of the VrgG family were identified. THSYN_15360 belongs to the vgr_GE type Rhs family proteins similar sequences found in β-proteobacterial family of the *Burkholderiaceae* whereas THSYN_32425 is conserved in γ-proteobacteria and contains a type IV Rhs element. Togheter, the secretion machinery allows strain Cad16^T^ to interact within the highly populated chemocline with up to 10^7^ bacterial cells per milliliter. The secretion and uptake mechanism may also play a key role in the cell-to-cell contact with *Desulfocapsa thiozymogenes**.*

#### Buoyancy regulation and chemotaxis

Strain Cad16^T^ can possibly regulate buyoncy by gas vesicles that are formed with the encoded structural gas vesicle proteins. Whereas GvpA proteins forms the vesicle core (THSYN_11790, THSYN_11825, THSYN_15290, THSYN_18705 and THSYN_31215), GvpFL (THSYN_11800 and THSYN_18685), GvpK (THSYN_11785) and GvpN (THSYN_11815 and THSYN_18695) further stabilize the structure. Proteins homologoues to the transcriptional regulatory factors GvrA (THSYN_11850) and GvrC (THSYN_11830) from the enterobacterium *Serratia* sp. ATCC 39006 are also found in Cad16^T^.

The diurnal and sesonal behavior of vacuolated *Chromatiaceae* has been described for different lakes [50, 51]. In strain Cad16^T^ a diguanylate cyclase (THSYN_19835) is found upstream the circadian clock genes *kaiCBB* (THSYN_19820–19,830). These genes act togheter [52] and may synchronize optimal flotation within the chemocline.

#### CRISPR-Cas systems

Bacterial CRISPR-Cas systems provide a mechanism against bacteriophage infection and plasmid transformation [53]. A CRISPR locus is composed out of a 300–500 bp leader sequence, spacer sequences (21–72 bp), complementary to foreign DNA, and direct repeats (DRs, 24–40 bp) flanking them [53–55]. Adjacent *cas* genes encode protein that are co-transcribed with the CRISPR locus and interfere with invading DNA guided by the specific spacers [56, 57].

Five CRISPR repeat regions (CRR1-CRR5) were identified in the genome of strain Cad16^T^, four being located on the chromosome and one on the plasmid pTs485 (Fig. 3). The number of DRs ranges from 19 (CRR4) to 146 (CRR2) as seen in Table 6.

**Table 6:** CRISPR-Cas loci detected in “T. syntrophicum*”* sp. nov. strain Cad16^T^ genome

Localization	Name	CRISPR start	CRISPR end	CRISPR length (bp)	DR consensus	DR length	No. of spacers	CRISPR-Cas loci^a^
chromosome	CRR1CRR1	1,879,131	1,881,639	2508	GCTTCAATGAGGCCGCGGCGAATTCGCCGCGGAAAC	36	34	type I-U
	CRR2CRR2	1,883,646	1,894,325	10,679	GCTTCAATGAGGCCGCGGCGAATTCGCCGCGGAAAC	36	146	
	CRR3CRR3	4,626,522	4,629,249	2727	GCATCGCCCGGCCAATTGGCCGGGCGCGGATTGAAAC	37	37	type I-C
	CRR4CRR4	5,078,034	5,085,199	7165	GTTTCCGCGGCGAATTCGCCGCGGCCTCATTGAAGC	36	98	–
pTs485	CRR5CRR5	391,741	393,104	1363	GTAGCGCTACTCCGAGCCGCAAAGGCTATTGAAAC	35	19	–

^a^ CRISPR-Cas classification according to Makarova et al. [58]

BLASTn analysis of the CRISPR DRsusing the CRISPRfinder platform revealed similarities in CRR1, CRR2 and CRR4 to sequences of “*T. mobilis**”* 8321 (57 hits, 2 mismatches) and “*Thioalkalivibrio sulfidophilus**”* HL-EbGr7 (63 hits, 3 mismatches). The DRs found in CRR3 are similar to the ones in *Halothiobacillus neapolitanus* c2 (31 hits, 4 mismatches), whereas the DRs in CRR5 are similar to the ones found in *Vibrio alginolyticus* NBRC 15630 (1 hit, 5 mismatches).

Furthermore, three CRISPR-Cas loci were identified in the strain Cad16^T^ sequence, containing *cas3* genes that are characteristic for type I CRIPSR-Cas systems [58]. A complete CRISPR-Cas loci (THSYN_08045–08070) is located 201 bp upstream of CRR2 and is assigned to subtype I-U, containing the signature protein (THSYN_08055) of the GSU0054 family (TIGR02165 and a *cas3*, THSYN_08070) with C-terminal HD domain (TIGR01596) [58]. Another CRISPR array (THSYN_19240–19,290) is located 182 bp upstream of CRR3 and is classified as subtype I-C due to the *cas8c* gene and the lack of a *cas6* sequence. Additionally, an incomplete CRISPR-Cas locus (CRR5) is identified on plasmid pTs485, encoding for Cas2, Cas1, (THSYN_19240–19,245, THSYN_19265, THSYN_19275, THSYN_19285 and THSYN_19,290).

## Conclusions

We report on the first complete genome sequence of “*Thiodictyon syntrophicum**”* sp. nov. strain Cad16^T^ and the metabolic versatility of this environmentally relevant organism. The observed carbon fixation potential can be explained by the highly developed photosynthesis machinery that is coupled to the sulfur and carbon metabolism. Within the changing conditions in the chemocline, strain Cad16^T^ is able to optimally use light, different organic and inorganic carbon compounds, reduced sulfur, nitrogen and oxygen. The two 0.4 Mb plasmids found in Cad16^T^ are unique for known PSB species and we report structural similarity to sequences from α- and γ-proteobacterial phototrophs. The availability of the complete genome sequence of strain Cad16^T^ will facilitate further studies that elucidate its role as key species of the chemocline and the tight association with the *Desulfocapsa* sp. and the interaction with different PSB and GSB species present in the anoxic part of Lake Cadagno. Due to the limited molecular data on other *Thiodictyon* strains and no reference strains available, no (digital) DNA-DNA hybridization experiments could be performed. However, the result from phylogenetic analyses on 16S rRNA sequence level, comparative genomic analyses as well a morphological and physiological differences (see above) indicate a novel species within the genus *Thiodictyon*.

The decribed isolate is therefore proposed as *“**Thiodictyon syntrophicum**”* sp. nov. strain Cad16^T^, a novel species within the genus *Thiodictyon*.

A formal description of the proposed novel species follow below:

**Description of “** *Thiodictyon syntrophicum* ***”*** **sp. nov.**

*“**Thiodictyon syntrophicum**”* (syn.tro’phi.cum. Gr. pref. Syn, together with; Gr. adj. *Trophikos*, nursing, tending or feeding; N.L. neut. Adj. *syntrophicum*, syntrophic).

Gram-negative, cells are oval-round shaped and 1.4–2.4 μm in diameter, non-motile, vacuolated and contain BChl *a* and okeneone. Growth as single cells, as well as in aggregates with up to 100 cells in a EPS layer. Assimilation of elemental sulfur in intracellular sulfur globules. Grow photoautotrophically in Pfennig's minimal medium with a doubling time of 121 h at 20–23 °C, a pH of 6.8–7.2, at 1 mM sulfide and a photoperiode of 12 h dark/ 12 h light. Dense cultures show a milky purple-red and milky color. Carbon assimilation via Calvin cycle. Following carbon substrates were utilized at a concentration of 5 mM: acetate, fructose and pyruvate. No growth was observed with 5 mM butyrate, ethanol, formate, fumarate, glucose, glycerol, lactate, malate, propanol, propionate and succinate, respectively. Chemolitoautotrophic growth was observed with 5% Oxygen and 0.02% hydrogen sulfid and 0.07% thiosulfate, or with 0.07% sulfide only, respectively.

The type strain Cad16^T^ (=JCM 15483^T^ =KCTC5955^T^) was isolated from a sulfidic chemocline in the alpine Lake Cadagno in Switzerland. The genome size of the type strain is 6.84 Mb (chromosome), contains two plasmids, pTs485 (0.49 Mb) and pTs417 (0.42 Mb) and the G + C content of the genome is 66.22%. The 16S RNA gene sequence of strain Cad16^T^ is deposited under the GenBank/EMBL/DDBJ accession number AJ511274. The complete genome sequence of the type strain Cad16^T^ is deposited under the GenBank ID CP020370, CP020371 and CP020372. The type strain has been deposited both at the Japan Collection of Microorganisms (JCM 15483^T^) and at the Korean Collection for Type Cultures (KCTC 5955^T^).

## Additional file

Additional file 1: **Figure S1.** Phylogenetic placement of “T. syntrophicum” strain Cad16^T^ within the other 12 Chromatiaceae species with a publicly available whole genome sequences. Additionally, the closely related phylogenetic lineages *Nitrosococcus*, Rheinheimera and Arsukibacterium are also included. Strain Cad16^T^ is most closely related to *L. purpurea* DSM 4197. The maximum likelihood tree was inferred from 100 concatenated single-copy orthologues sequences [61] and a total of 1000 bootstrap replicates were performed. Numbers at the nodes indicate the SH-aLRT support (%) and ultrafast bootstrap support (%). OrthoMCL [64], was used to define at set orthologues proteins between these 23 species. Hundred single-copy orthologues were randomly chosen and aligned with MUSCLE [66] . The best-fit phylogenetic model and subsequent consensus tree computation, based on maximum-likelihood and 1000 bootstrap iterations, was performed with the *IQ-TREE* software [62]. Nodes with both, 100% SH-aLRT and ultrafast bootstrap support, are indicated with filled black circle symbols for convenience. (TIF 57220 kb)

## Abbreviations

ABC: ATP-binding cassette
APS: adenosine-5′-phosphosulfate
BChl *a*: bacteriochlorophyll *a*
Cas: CRISPR assosiated
CBB: Calvin–Benson–Bassham cycle
CRISPRs: clustered regularly interspaced short palindromic repeats
DC/HB: dicarboxylate/4-hydroxybutyrate cycle
DR: direct repeat
*dsr*: dissimilatory sulfite reductase
EPS: extracellular polymeric substances
GSB: green sulfur bacteria
HiPIP: high-potential iron-sulfur protein
PGC: photosynthesis gene cluster
PSB: purple sulfur bacteria
RC: reaction center
Rubis-CO: ribulose-1,5-bisphosphate carboxylase/oxygenase
SGB: sulfur globule
SMRT: single molecule real-time
SRB: sulfur reducing bacteria
T4P: type IV pilus
T6SS: type VI protein secretion system
TAT: twin-arginine translocation
TCA: tricarboxylic acid cycle
TRAP: tripartite ATP-independent periplasmic
