# Supplementary figures and images for: Complete genome sequence of “Thiodictyon syntrophicum” sp. nov. strain Cad16T, a photolithoautotrophic purple sulfur bacterium isolated from the alpine meromictic Lake Cadagno
Source: Stand Genomic Sci. 2018 May 9;13:14. doi: 10.1186/s40793-018-0317-z (PMC5944118; doi:10.1186/s40793-018-0317-z)

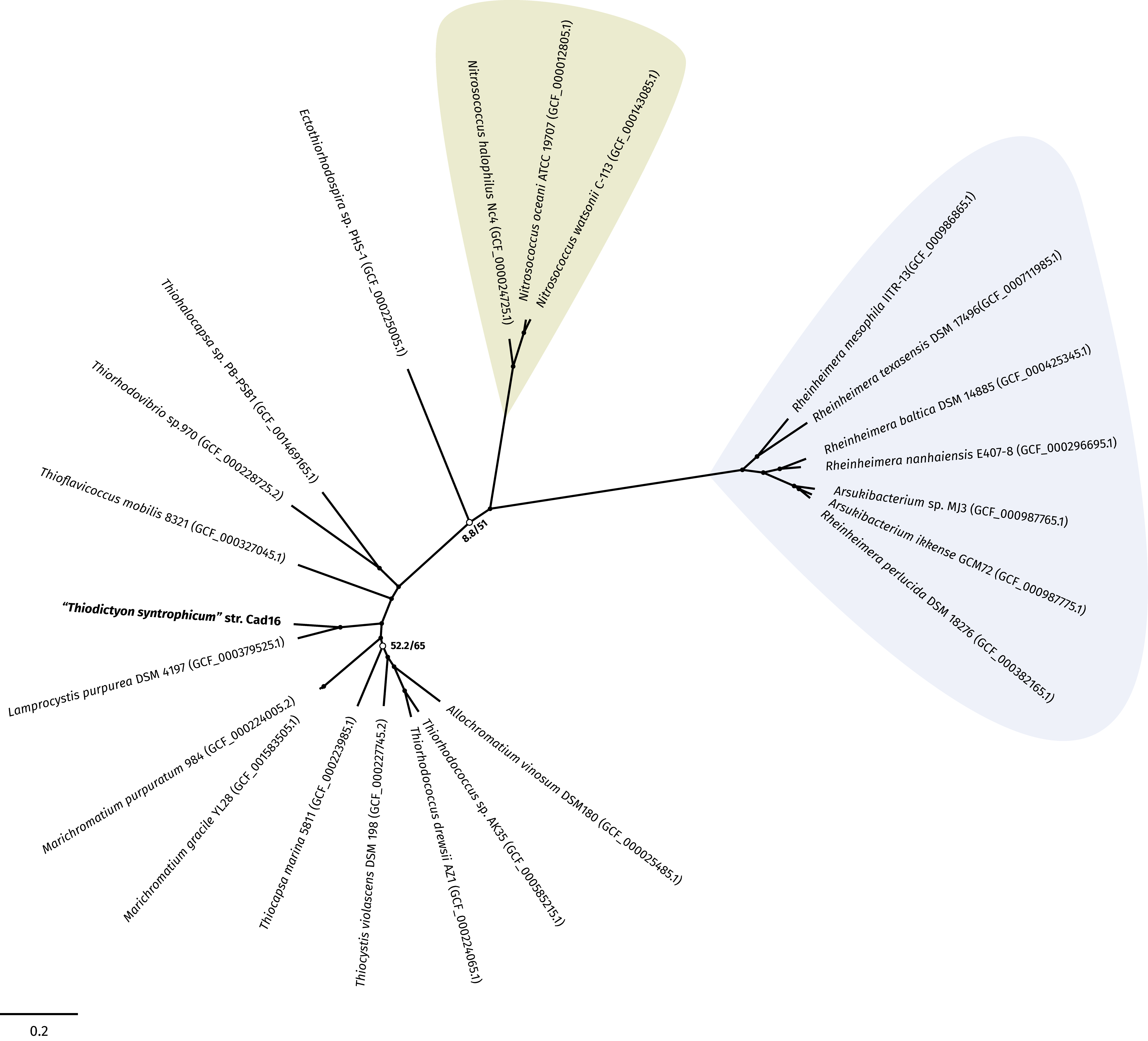

Supplement: Supplementary file 1 — Figure S1. Phylogenetic placement of “T. syntrophicum” strain Cad16T within the other 12 Chromatiaceae species with a publicly available whole genome sequences. Additionally, the closely related phylogenetic lineages Nitrosococcus, Rheinheimera and Arsukibacterium are also included. Strain Cad16T is most closely related to L. purpurea DSM 4197. The maximum likelihood tree was inferred from 100 concatenated single-copy orthologues sequences [61] and a total of 1000 bootstrap replicates were performed. Numbers at the nodes indicate the SH-aLRT support (%) and ultrafast bootstrap support (%). OrthoMCL [64], was used to define at set orthologues proteins between these 23 species. Hundred single-copy orthologues were randomly chosen and aligned with MUSCLE [66] . The best-fit phylogenetic model and subsequent consensus tree computation, based on maximum-likelihood and 1000 bootstrap iterations, was performed with the IQ-TREE software [62]. Nodes with both, 100% SH-aLRT and ultrafast bootstrap support, are indicated with filled black circle symbols for convenience. (TIF 57220 kb) [file 40793_2018_317_MOESM1_ESM.tif]
